# Novel color via stimulation of individual photoreceptors at population scale

**DOI:** 10.1126/sciadv.adu1052

**Published:** 2025-04-18

**Authors:** James Fong, Hannah K. Doyle, Congli Wang, Alexandra E. Boehm, Sofie R. Herbeck, Vimal Prabhu Pandiyan, Brian P. Schmidt, Pavan Tiruveedhula, John E. Vanston, William S. Tuten, Ramkumar Sabesan, Austin Roorda, Ren Ng

**Affiliations:** ^1^ Department of Electrical Engineering & Computer Sciences, University of California, Berkeley, Berkeley, CA 94720, USA.; ^2^ Herbert Wertheim School of Optometry & Vision Science, University of California, Berkeley, Berkeley, CA 94720, USA.; ^3^ Department of Ophthalmology, University of Washington School of Medicine, Seattle, WA 98195, USA.

## Abstract

We introduce a principle, Oz, for displaying color imagery: directly controlling the human eye’s photoreceptor activity via cell-by-cell light delivery. Theoretically, novel colors are possible through bypassing the constraints set by the cone spectral sensitivities and activating M cone cells exclusively. In practice, we confirm a partial expansion of colorspace toward that theoretical ideal. Attempting to activate M cones exclusively is shown to elicit a color beyond the natural human gamut, formally measured with color matching by human subjects. They describe the color as blue-green of unprecedented saturation. Further experiments show that subjects perceive Oz colors in image and video form. The prototype targets laser microdoses to thousands of spectrally classified cones under fixational eye motion. These results are proof-of-principle for programmable control over individual photoreceptors at population scale.

## Teaser

Image display by cell-by-cell retina stimulation, enabling colors impossible to see under natural viewing.

## INTRODUCTION

**Fig. 1.**
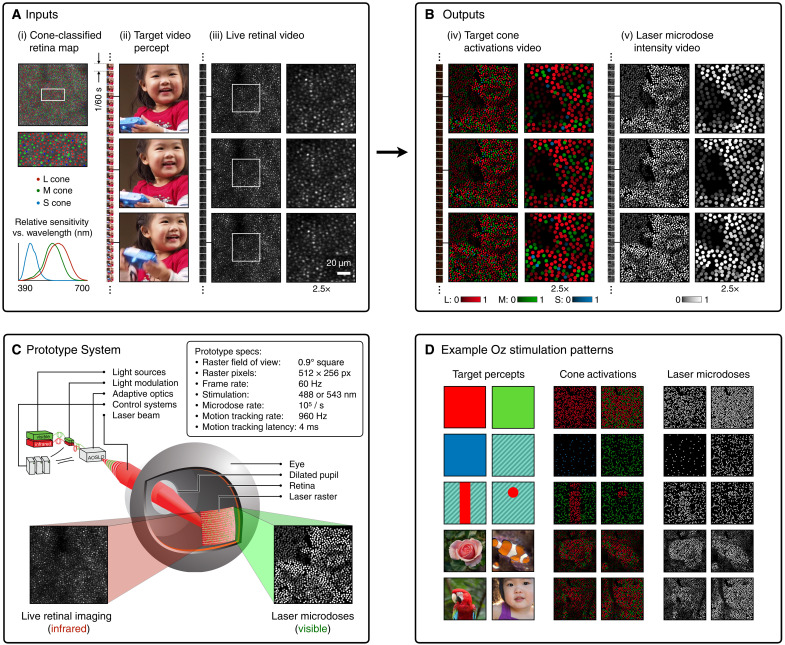
Overview of principle and prototype system. (**A**) System inputs. (i) Retina map of 10^3^ cone cells preclassified by spectral type (*7*). (ii) Target visual percept (here, a video of a child, see movie S1 at 1:04). (iii) Infrared cellular-scale imaging of the retina with 60-frames-per-second rolling shutter. Fixational eye movement is visible over the three frames shown. (**B**) System outputs. (iv) Real-time per-cone target activation levels to reproduce the target percept, computed by: extracting eye motion from the input video relative to the retina map; identifying the spectral type of every cone in the field of view; computing the per-cone activation the target percept would have produced. (v) Intensities of visible-wavelength 488-nm laser microdoses at each cone required to achieve its target activation level. (**C**) Infrared imaging and visible-wavelength stimulation are physically accomplished in a raster scan across the retinal region using AOSLO. By modulating the visible-wavelength beam’s intensity, the laser microdoses shown in (v) are delivered. Drawing adapted with permission [Harmening and Sincich (*54*)]. (**D**) Examples of target percepts with corresponding cone activations and laser microdoses, ranging from colored squares to complex imagery. Teal-striped regions represent the color “olo” of stimulating only M cones.

We introduce a new principle for displaying color, which we call Oz: optically stimulating individual photoreceptor cells on the retina at population scale to directly control their activation levels. In principle, arbitrary colored visual imagery can be displayed by this cell-by-cell approach, but doing so requires exquisite precision in reproducing the dynamic stimulation levels at each photoreceptor as imagery traverses the retina under eye movements (see Fig. 1). As proof of principle, we perform human subject experiments on a prototype Oz system that stimulates thousands of retinal cone cells.

**Fig. 2.**
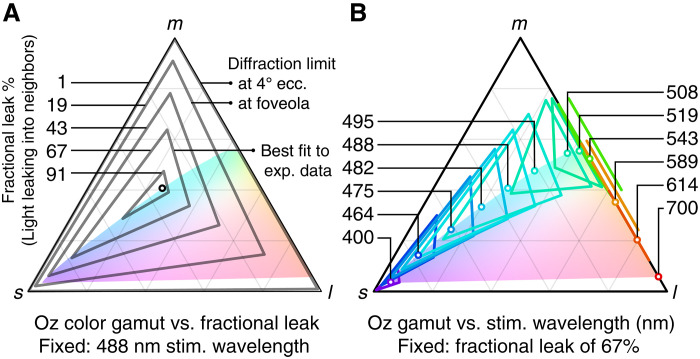
Theoretical model of Oz color gamut as a function of fractional leak and stimulation wavelength. (**A**) Gamut shrinks from the full *lms* chromaticity triangle to the stimulation wavelength (open circle) as the fractional light leak grows; note that this fraction depends on the intercone spacing, which varies across the retina. The colored region is the gamut of natural human colors. (**B**) Gamut varies in chromaticity, position, and shape as a function of stimulation wavelength. For readability, extra copies of the gamuts for 543 and 589 nm are drawn next to the *lm* edge.

Theoretically, Oz enables display of colors that lie beyond the well-known, bounded color gamut of natural human vision (*1*). In normal color vision, any light that stimulates an M cone cell must also stimulate its neighboring L and/or S cones, because the M cone spectral response function lies between that of the L and S cones and overlaps completely with them (*2*, *3*). However, Oz stimulation can by definition target light to only M cones and not L or S, which in principle would send a color signal to the brain that never occurs in natural vision. Theoretically, Oz expands the natural human color gamut to any (L, M, and S) color coordinate (see Fig. 2). In practice, we achieve a partial expansion of colorspace toward this theoretical maximum.

The closest prior art for selectively exciting M cones is targeting light to only one (*4*–*7*) or two (*8*) cones at a time. Aside from cone-targeted methods, the only other methods to selectively excite M cones use visual pre-adaptation such as bleaching L photopigment with red light before displaying green light (*9*, *10*). However, such percepts rely on fleeting adaptation states and after-images, so they are difficult to measure precisely (*9*, *11*). A different method called silent substitution (*12*, *13*) can isolate activation changes to M cones, but requires baseline activation of the other cone classes and cannot display colors beyond the human gamut. In contrast to these approaches, our Oz prototype displays colors beyond the natural human gamut over a large enough area for color matching, for sustained durations, and within arbitrary colored imagery.

Our Oz prototype is a proof-of-principle that builds upon the cone-targeted methods (*4*–*8*) that use adaptive optics scanning light ophthalmoscopy (AOSLO) (*14*). First, adaptive optics optical coherence tomography (AO-OCT) (*15*, *16*) is used to spectrally preclassify the LMS type of 10^3^ retinal cone cells (*17*) per subject. Then, AOSLO produces Oz percepts by imaging the retina in infrared to near-invisibly track eye motion at cellular scale, and targeting 10^5^ visible-wavelength laser microdoses per second to each cone cell. The visual field of view of the prototype is a 0.9° square centered at 4° adjacent to a gaze-fixation target.

**Fig. 3.**
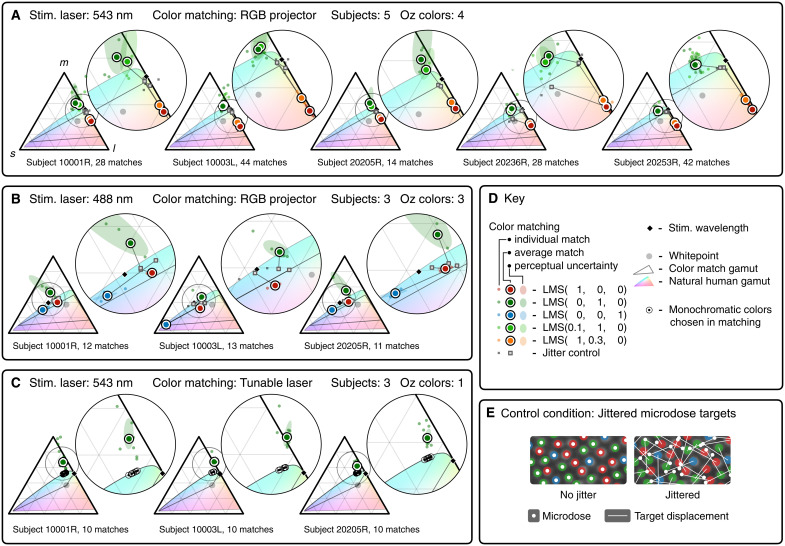
Color matching of Oz colored squares produced by cone-by-cone stimulation. (**A** to **D**) Each *lms* chromaticity triangle plots color matches for one subject with the indicated stimulation wavelength and type of matching color system (RGB projector, or tunable near-monochromatic laser and projector white). Target colors are specified as (L, M, and S) triplets, which are the relative light intensity levels directed to each cone class. Color matches to different target colors are denoted with differently colored markers. Each triangle also plots: color matches for randomly interleaved jitter control condition [see (E) and the “Design of prototype” section]; coordinates of the stimulation wavelength; natural color gamut of human vision; gamut of the matching color system and its whitepoint; and perceptual uncertainty ellipses for the average color matches (projected JND ellipsoid at the coordinates of the “positive” component of the color match, computed from CIELAB/${\Delta E}_{ab}^{*}$, scaled three times the actual size; see the “Plotting perceptual uncertainty in matching” section in Materials and Methods). Ellipses not visible are smaller than their associated markers. (**E**) Illustration of the control condition randomly interleaved into all experiments: microdose target locations are randomly jittered by two intercone spacings in Oz stimuli that are otherwise identical to the experimental condition.

We mapped the empirical colorspace coordinates of Oz colors in practice using formal color matching experiments (Fig. 3) and collected qualitative judgments of hue and saturation. These experiments confirmed that the prototype successfully displays a range of hues in Oz: e.g., from orange to yellow to green to blue-green with a 543-nm stimulating laser that ordinarily looks green. Further, color matching confirms that our attempt at stimulating only M cones displays a color that lies beyond the natural human gamut. We name this new color “olo,” with the ideal version of olo defined as pure M activation. Subjects report that olo in our prototype system appears blue-green of unprecedented saturation, when viewed relative to a neutral gray background. Subjects find that they must desaturate olo by adding white light before they can achieve a color match with the closest monochromatic light, which lies on the boundary of the gamut, unequivocal proof that olo lies beyond the gamut.

**Fig. 4.**
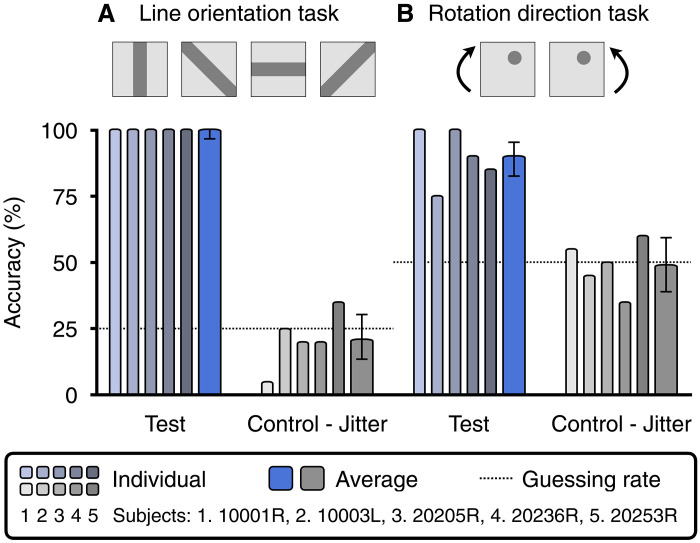
Image and video recognition experiments. We tested subjects’ ability to recognize image and video content consisting of Oz colors: (**A**) a 4-alternative forced choice (4-AFC) line orientation recognition task, and (**B**), a 2-AFC rotation direction task experiments. Oz stimuli consisted of equiluminant red lines and disks presented on an olo background, as depicted. The bar graphs show individual subject performance over 20 trials per condition and average accuracy across five subjects with 95% confidence intervals. In experimental conditions with Oz microdoses delivered experimentally (blue bars), subjects are able to accurately identify line orientation and rotation direction. In control-group stimuli (gray bars), where cone-targeting is compromised through jittering microdose target locations, task accuracy is reduced to guessing rate as indicated by the dashed lines.

In control experiments, Oz color matches “collapse” to the natural color of the laser, as expected, if we “jitter” the target location of each laser microdose so that it incorrectly lands on a random neighboring cell. In addition, subjects clearly perceive Oz hues in image and video form, such as an oriented red line or a rotating red dot on an olo background (Fig. 4), and cannot do so under the jitter control condition.

For any color distinct from the natural color of the stimulating laser to be perceived in Oz, our prototype system must perform high-resolution retinal imaging, high-speed tracking of eye motion, and low-latency stimulus delivery (*18*). Demonstrating colors outside of the natural human gamut in Oz is the perceptual signature that each of these system components is operating successfully in unison. This technical achievement introduces an experimental platform for visual perception with a new class of precision, programmable control, and cellular scale.

## RESULTS

### Theory of cell-by-cell color

We plot the colors generated by our Oz prototype on a Maxwell triangle (*19*) with barycentric coordinates $\left(l,m,s\right)=\frac{\left(L,M,S\right)}{L+M+S}$. This triangle displays the chromaticity (hue and saturation of a color) in two dimensions (2D), while projecting out its total activation (*L* + *M* + *S*). In these diagrams, the color-filled subregion plotted at the bottom is the natural human gamut, which spans all chromaticities achievable via ordinary spectral mixtures of light.

In theory, the full area of the chromaticity triangle itself is the fundamentally larger color gamut that is accessible via cell-by-cell stimulation in Oz, assuming idealized conditions that produce perfect localization of light to target cones. In practice, however, a fraction of the light will miss target cones and stimulate neighboring cells, causing the resulting activation pattern to shift from the intended Oz color toward the laser’s natural color.

The effect of such stray cone activation on achievable colors is predicted in Fig. 2. The key factors are: the point-spread function (PSF) of the laser microdoses on the retina relative to the spacing of cone cells, the cone’s spatial light gathering function (*20*–*22*), errors in microdose targeting during eye movement, the retina’s L:M:S cone proportion, and the stimulating wavelength (details in the “Theoretical modeling of Oz color gamuts” section in Materials and Methods).

Figure 2A illustrates how fractional light leak would affect the gamut of achievable Oz chromaticities. In theory, a diffraction-limited PSF would enable Oz to address nearly all possible chromaticities in the *lms* triangle when stimulating the retina at 4° eccentricity, as shown, but not in the foveola where cone cells are smallest. In practice, the total leakage of light includes more than diffraction due to factors such as residual aberrations after adaptive optics focusing and errors in microdose targeting due to computational latency during eye movement. Measuring these factors directly is challenging, but a best fit of the model shown in Fig. 2A against the experimental color matching data in the upcoming “Color matching experiments” section suggests that, of the light captured by cones, one-third is confined to the target cell and two-thirds is captured by neighboring cones. Despite this unintended light leak, this level of accuracy succeeds in displaying color beyond the natural human gamut in our Oz prototype.

Figure 2B illustrates how the stimulating wavelength would affect the gamut of achievable Oz chromaticities. The shape of this gamut reflects the relative responses of the L, M, and S cone cells at a given stimulating wavelength, forming a triangle, line, or single point depending on the number of cone types that respond at that wavelength.

### Design of prototype

We build our Oz prototype on an AOSLO (*14*) that simultaneously images and stimulates the retina with a raster scan of near-diffraction-limited laser light over a 0.9° square field of view. Using nearly invisible infrared light to image the retina, we can track the eye’s motion in real time. We compensate for this motion and deliver pulses of visible-wavelength laser light dynamically targeted at each cone cell within the field of view. These laser microdoses are delivered at a rate of 10^5^ per second to a population of 10^3^ cones.

To achieve an intended LMS activation through cone-targeted stimulation, the spectral type of each cone must be known. In a preparatory step, cone cells are classified by spectral type in the subject’s retina using recently developed optoretinography techniques in an AO-OCT system (*15*, *23*). In this study, we use a classified region containing 1000 to 2000 cones located near 4° eccentricity from the foveola.

We show Oz stimuli to human subjects and perform the following experiments: color matching of uniform Oz color squares and image/video recognition experiments. All Oz stimuli are presented within the 0.9° square field of view, 4° adjacent to a gaze fixation point, so that the stimulated area falls within the classified region of retina. As a control condition, stimuli are randomly repeated with microdose delivery intentionally compromised. During these control trials, each microdose is “jittered” randomly so that it lands two cones away from the target.

### Color matching experiments

We conduct color matching experiments to formally measure the chromaticity coordinates of Oz colors. Two different stimulation wavelengths are tested: 488 nm, which can activate all three L, M, and S cone types, and 543 nm, which is near the peak of L and M, and only minimally activates S. We use two different color matching systems: first, a red-green-blue (RGB) projector, and second, a near-monochromatic laser of tunable wavelength that can be mixed with white projector light. The latter can produce colors that lie on the edge of the natural human gamut, eliminating ambiguity as to whether our attempts to display olo truly lie beyond the natural human gamut. During a color matching trial, the subject sees 0.9° squares of Oz and controllable color, coincident in space and alternating in time, so that subjects must judge match equality using the same patch of retina, eliminating effects from differences in adaptation across the retina. As usual with color matching (*1*), subjects can add light to the Oz color (so-called “negative” light) if necessary to achieve an exact color match; its color coordinates are subtracted from those of the controllable square to calculate the matched color. Subjects are also prompted to qualitatively name the hue and rate the saturation (scale of 1 to 4) of the squares of Oz color and controllable color.

Figure 3 graphs results of the color matching experiments. Five subjects performed 222 color matches. We highlight four observations.

First, Oz colors form a triangle around the stimulation wavelength for 488 nm (Fig. 3B), and a line of colors for 543 nm (Fig. 3A), consistent with theory in the “Theory of cell-by-cell color” section. Second, the jitter control condition causes the color to “collapse” toward the stimulation wavelength, as expected.

Third, the variance in matching *lms* chromaticity increases with the distance of Oz colors from the gamut of the color matching system. This trend is consistent with the geometric analysis in the “Plotting perceptual uncertainty in matching” section in Materials and Methods explaining why perceptual uncertainty in chromaticity increases when light must be added to the test color to achieve a match.

Fourth, Fig. 3C provides unequivocal confirmation that olo lies beyond the natural human gamut. In these matches, all subjects found it necessary to desaturate olo with projector white in order to match the (near) monochromatic colors shown, which lie on the boundary of the natural human gamut. These matching monochromatic wavelengths, from 501 to 512 nm, are the most saturated teal hues for normal color vision under the test subjects’ viewing conditions.

Subjects’ qualitative hue naming and saturation ratings corroborate these quantitative results, although the Abney effect [a shift in hue with saturation (*24*)] opens the possibility that the hue of the wavelength at best match may not exactly represent the hue of the undiluted olo color. Color names volunteered for olo include “teal,” “green,” “blue-greenish,” and “green, a little blue.” Subjects consistently rate olo’s saturation as 4 of 4, compared to an average rating of 2.9 for the near-monochromatic colors of matching hue shown in Fig. 3C.

### Image and video recognition experiments

We design image and video recognition experiments to probe the ability of human subjects to understand images rendered in Oz. We use four-alternative forced choice (4-AFC) and 2-AFC tasks where subjects can only succeed using hue information created through accurate Oz stimulation. In the 4-AFC task, subjects must identify the orientation of a line in an image. In the 2-AFC task, subjects must detect the rotation direction in a video of a moving disk. In these stimuli, the lines and disks are rendered as red (all-L cone) on an olo background (all-M cone), delivered using a stimulating wavelength of 543 nm. A calibration step is performed to ensure that the foreground and background are equiluminant (see the “Image and video recognition experiments” section in Materials and Methods), so that in the jitter control condition, all hue and luminance cues are removed and the task reduces to guessing.

Figure 4 plots the results of the 4-AFC line orientation task and the 2-AFC rotation direction task. In the experimental condition, subjects are able to reliably detect both line orientation and motion direction (blue bars). In the jitter control condition, subjects’ performance is reduced to guessing for both tasks (gray bars). Qualitatively, subjects report seeing red or orange lines and disks on a blue-green or green background, when the task was easy, compared with a yellow-green square, when they were forced to guess. The former correlates directly with accurate Oz microdose deliveries, and the latter with the jitter control condition, where only the natural color of the 543-nm light should be perceived.

## DISCUSSION

All color reproduction technology today, including RGB displays and CMYK printers, is based on spectral metamerism, producing light of a spectral power distribution that causes the same activation level as a target color for each cone type in the retina. This approach dates back to at least 1861, when Maxwell gave a live demonstration at the Royal Institution of superimposing red, green, and blue images to produce the appearance of full-color images to human observers (*25*).

The Oz principle of color reproduction introduced in this paper is fundamentally different, and can be thought of as spatial metamerism in the sense that it is based on shaping the spatial distribution of light on the retina rather than its spectral distribution. Unlike conventional metamerism that requires at least three light primaries, we showed that spatial metamerism can produce a range of colors from a single monochromatic light (e.g., 543 nm laser). In addition, spatial metamerism enables fundamentally new colors, such as olo, that cannot be produced by conventional metamerism.

The required control of photoreceptor activations at population scale is technically challenging, and our experiments are limited to a 0.9° square field of view centered at 4° eccentricity, which requires gaze fixation. Enlarging Oz to an apparent N° square field-of-view and allowing subjects to gaze freely presents substantial technical challenges. It would require spectral classification of the central 2N° square patch of retina [classification has progressed to within 0.3° eccentricity, but not yet to the smallest cells in the fovea (*23*, *26*)]. It would require improving optical focus and spatiotemporal accuracy to achieve diffraction-limited microdoses within each cell, while allowing saccadic eye motion within the video field of view. It would also require scaling up; for example, to 4·10^4^ cones and 10^7^ microdoses per second for a 2° × 2° “free-gaze” Oz system.

Spatial metamerism requires highly dynamic spatiotemporal patterns of activation on the retina. For example, viewing a uniform square of color, corresponding to a constant ratio of L, M, and S activation, actually represents dynamic switching on and off of each cone’s activation as it enters and exits the boundary of the square during fixational eye drift (e.g., see movie S1 at 1:55). In our color matching experiments, such switches in activation occur on the order of 1000 times per second. In contrast, simply stabilizing an unchanging activation level at all cones results in the color percept rapidly fading to become invisible (<10 s), consistent with well-known Troxler fading. The dynamism of the spatiotemporal pattern of activation increases markedly when considering general image and video percepts (e.g., see movie S1 at 1:04), where fine image details move across a cone during eye movement and cause activation levels to fluctuate on the order of 10^5^ times per second across the stimulation area. Reproducing such patterns in Oz requires fine-grained and complex programmability of each cell’s microdose intensity, and can be thought of as extending computer graphics and virtual reality from screen pixels down to the level of individual photoreceptors.

Oz represents a new class of experimental platform for vision science and neuroscience, which strives for complete control of the first neural layer to the brain, programmability of every photoreceptor’s activation at every point in time. Our prototype is an advance toward this class of neural control, and we demonstrate its ability to accurately deliver microdoses to target cones despite the challenges presented by constant fixational eye motion and the optical aberrations of the eye. When Oz microdoses are intentionally “jittered” by just a few microns, subjects perceive the stimulating laser’s natural color. When these same Oz microdoses are delivered accurately, subjects can be made to perceive different colors of the rainbow, unprecedented colors beyond the natural human gamut, and imagery like brilliant red lines or rotating dots on an olo background.

This new class of programmable platform will enable diverse new experiments. For example, Oz can support systematic probing of phenomena such as the threshold at which a small number of cones begin to contribute to a stable color percept (*4*, *5*, *7*, *27*, *28*), or the nonlinear function of a retinal ganglion cell’s response to cone activations in its receptive field (*29*, *30*). Oz can reproduce and then enable programmable “micro-adjustments” to probe the cone activations underlying visual phenomena that operate near the limits of visual perception, such as the two colored-line illusion (*31*) or visual loss with high levels of cone dropout (*32*, *33*). More ambitiously, Oz can be programmed to probe the plasticity of human color vision. For example, gene therapy has been used to add a third cone type in adult squirrel monkeys, producing trichromatic color vision behavior (*34*). Analogously, Oz can program signals to the human brain as if a subset of cones were filled with a new photopigment type, allowing for probing of the qualitative color experience which could not be revealed by the results of the study done in squirrel monkeys. Such an approach can flexibly probe neural plasticity to boosting color dimensionality (*35*) in humans, such as attempting to elicit full trichromatic color vision in a red-green colorblind person, or eliciting tetrachromacy in a human trichromat.

## MATERIALS AND METHODS

### Human subjects

Five subjects were recruited for this experiment [subject number, age, sex, L:M:S ratio, center-to-center cone spacing at 4°]: [10001R, 40, M, 60:32:8, 1.6′], [10003L, 57, M, 58:36:6, 1.7′], [20205R, 44, M, 62:30:8, 1.6′], [20236R, 42, M, 61:30:9, 1.8′], and [20253R, 30, F, 62:30:8, 1.5′]. All subjects self-reported as having normal color vision and no ocular disease condition. Subjects 10001R, 10003L, and 20205R are coauthors on the paper and were blinded to the test conditions but were aware of the purposes of the study. The other two subjects were members of the participating lab at the University of Washington but were naive to the purposes of the study. The studies were approved by the institutional review boards at the University of California, Berkeley (2020-02-12997) and the University of Washington (STUDY00013473). We obtained informed consent from all participants.

### Theoretical modeling of Oz color gamuts

The “Theory of cell-by-cell color” section presents a model of achievable Oz color gamut as a function of the fraction of light leaking into neighboring cells rather than the target cell. The model predicts the perceived LMS value given an input microdose wavelength, target LMS value, and subject cone ratio. We work in the 3D LMS coordinates defined by projection of spectral power distribution functions against the Stockman and Sharpe human cone responses (*2*, *3*). We plot colors on the *lms* chromaticity triangle, with barycentric coordinates $\left(l,m,s\right)=\frac{\left(L,M,S\right)}{L+M+S}$.

Fractional leak is assumed to collect in neighboring L, M, and S cones in proportion to their relative frequency in the subject’s retina (reported per subject in the “Human subjects” section, and Fig. 2 is for subject 10001R). For example, if the fractional leak is 60%, the model deposits 40% of the light from each microdose into the target cone and distributes the remaining 60% of the light uniformly into all other cones. The activation at each cell is a product of the total light received and the sensitivity of that cone to the light’s wavelength. The model’s predicted LMS value is computed as the average L, M, and S cone cell activation across the population of cells.

Idealized, diffraction-limited performance is shown in Fig. 2A, and varies with retinal eccentricity because of increasing spacing of cones (*36*). The fractional leak is computed by assuming that each microdose is perfectly centered on its target cone cell, that adaptive optics achieves a diffraction-limited optical point-spread-function with a dilated 6-mm pupil, modeling a hexagonal packing of cone cells, and modeling the spatial light gathering function of each cone cell as equal to a Gaussian with a full-width-half-maximum that is half the cone inner segment diameter (ISD) (*22*). The 4° and foveola regions are modeled with center-to-center spacings of 1.6′ and 0.4′, respectively, consistent with Curcio *et al.* (*36*) (using the observed per-subject center-to-center spacings reported in the “Human subjects” section yields no substantial difference on predicted idealized performance). The ISD is assumed to be a fraction of the spacing: two-thirds at 4° and equality in the tightly packed foveola.

In Fig. 2A, we also highlight the fractional leak that best fits the empirical color matching data. We compute a least-squares fit of the fractional leak parameter, to best explain the shift between target LMS values and matched LMS values in the experimental color matching data in the “Color matching experiments” section. It is important to compute the fit in the linear space of 3D LMS values, not in the projected 2D chromaticity space. In summary, this fit minimizes the root mean squared error (RMSE) in Cartesian LMS coordinates between the experimental data and our model’s output. The fit works as follows. For each experimental color match datum, we take the LMS target value and use the model to compute a predicted LMS value under a given fractional light leak. We compare the difference (modeling error) between this predicted LMS value and the subject’s experimental color match LMS value for that datum. We sum the errors for all the color match data in an RMSE sense. Then, we use a least squares solver to compute the fractional light leak value that minimizes the total modeling error. One practical detail is that there is a perceptual scaling factor between the predicted LMS value and the experimental LMS value, which differs for each experimental session, stimulation laser and cone class. These scaling factors are not known a priori but are inherent in the experimental match data; they represent unmeasured normalization of laser power across sessions and variation in individual subject cone response functions at the target laser powers. In the least squares solver, these scaling factors are variables (19 total), and we solve for the scaling factors and global fractional light leak that jointly minimize the total modeling error over all non-control color matches (190 total).

### Prototype system hardware

We built on an AOSLO platform described in previous publications (*14*, *37*). In this study, we used four spectral channels: a 940-nm channel for wavefront sensing, an 840-nm channel for retinal imaging, a 543-nm channel for retinal stimulation, and a blue channel configurable either as a 488-nm channel for retinal stimulation, or as a wavelength-tunable monochromatic source for matching use. Laser sources of the 940-, 840-, and 543-nm channels are drawn from the broadband spectral output of a supercontinuum laser (EXR-15, NKT, Birkerød, Denmark). The laser source of the blue channel comes from a separate supercontinuum laser (FIU-15, NKT, Birkerød, Denmark) passed through a tunable filter (VARIA, NKT, Birkerød, Denmark).

All channels (except the 940-nm channel) are passed through individually fiber-coupled acousto-optic modulators (AOMs) (Brimrose Corporation, Sparks, MD) that can modulate laser intensity up to 50 MHz, and are coaligned to make the pupil-conjugate planes optically coincident for each channel pair, with respective beam vergence precompensated to be opposite to the longitudinal chromatic aberration of a typical human eye (*38*). These adjustments ensure that all wavelengths are focused approximately at the same axial retinal depth. At the eye station, the laser powers in all channels are eye-safe, and are measured and recorded before every session.

In experiments, a custom-built Shack-Hartmann wavefront sensor operating with 940-nm light enables adaptive optics correction of eye aberrations in real time using a deformable mirror (DM97, ALPAO, Montbonnot-Saint-Martin, France), to achieve near-diffraction-limited focus at the photoreceptor layer of the retina, for all wavelength channels. We dilate and cyloplege our subjects using eye drops (1% tropicamide and 2.5% phenylephrine) to enable imaging through the largest possible pupil size (highest numerical aperture) and use adaptive optics to measure and correct for the aberrations of the eye and strive for near-diffraction-limited performance (*39*).

This laser spot is scanned in a raster pattern over a 0.9° square field of view using orthogonally oriented resonant and galvo mirrors, with a frame resolution of 512 × 256 pixels and a frame rate of 60 Hz. Light scattered from the retina is descanned and spectrally redirected to confocal pinholes mounted to individual photomultiplier tubes (PMTs) for each wavelength channel.

A custom-built field-programmable gate array (FPGA) board [initially in (*40*)] digitizes and aggregates all PMT signals into 512 × 16 pixel strips of each frame at 960 Hz as a rolling shutter video stream into a graphics processing unit (GPU) desktop computer that computes cone-by-cone microdose targets (see the “Prototype system software” section). The FPGA receives rasterized 14-bit stimulation signals from the desktop to drive AOMs that modulate the visible-wavelength laser intensities and deliver the intended microdoses of laser light to real-time cone positions.

In addition, a separate optically coaligned pathway, similar to the one used in (*41*), is used, which incorporates a projector display for showing fixation points and color matching targets, and a pupil camera for real-time pupil tracking.

### Prototype system software

#### Creating the spectrally classified retina map

We create a 1.8° square retina map for the subject (Fig. 1A) comprising a composite infrared image of the retina with metadata for the location of each cone and its spectral type. Although cone positions and types are stable, the reflectance appearance of different cones will change over time. We construct the composite retina image by first acquiring a set of 3 × 3 infrared AOSLO videos of the subject’s retina, with fields of view that overlap by 50% each to cover the desired area. We extend a global optimization algorithm, R-SLAM (*42*), to jointly solve for the composite retina image from the overlapping videos, as well as distortion corrections in the sinusoidal velocity of the resonant mirror. We use the Retina Map Alignment Tool [described in (*26*)], to align and copy over metadata about each cone’s location and spectral type from a master retina map for the subject. This master map of spectral classification is created once per subject through optoretinography on a separate AO-OCT instrument (*23*).

#### Eye motion tracking

We computationally track eye gaze translation and torsion with subcellular accuracy, by comparing the incoming 960-Hz stream of video strips against the retina map. We build on the normalized cross-correlation (NCC) strip-based matching method (*43*). We reduce false NCC matches using RANSAC (*44*), with full implementation detailed in (*45*). We add measurement of torsion by separately tracking the left and right halves of each incoming strip, fitting a matrix of rotation and translation to the midpoints of the independently tracked halves (*43*). This tracking algorithm runs on average in 0.8 ms on a GPU (GeForce RTX 3090, NVIDIA, Santa Clara, CA).

#### Computing target cone activations and laser microdose intensities

As shown in Fig. 1, we render the target video into a dynamic stream of target cone activations and laser microdose intensities. First, in a preprocessing step, we convert the RGB video pixels into LMS colorspace and spatially downsample to 64 × 64 pixel resolution to approximately match the spatial resolution of the cone mosaic within the system field of view.

During Oz stimulation, we render target video pixels into target cone activations. We store the cone target activations as a piece of metadata for each cone cell in the retina map. At the frame rate of the video, we continuously increment the target activation of all cones that fall within the bounds of the video at that instant, according to the real-time eye motion tracking. The activation increment for each cone is equal to the LMS video pixel value at the real-time location of the cone, taking the value in the LMS color channel that matches the spectral type of the cone.

In parallel computation, the desktop system rasterizes cone target activations into laser raster pixel values. Rasterization clears cone target activations by zeroing them out after instructing the FPGA to illuminate the laser raster pixel that will send a physical microdose of light to the cone’s real-time position on the retina. Rasterization is implemented by just-in-time computation and transmission to the FPGA of the 512 × 16 strip of raster pixel values that it will then stream to the laser modulation units. The real-time location of the current strip is defined by eye motion tracking and the offset of the strip within the video frame. For each cone on the retina map that is contained within the real-time location of the current strip, the cone’s position within the pixel array is located, and the corresponding pixel is set to the desired laser microdose intensity. This intensity value is equal to the target activation multiplied by the relative power of the laser, divided by the spectral response of that type of cone at the wavelength of the laser. During rasterization, microdoses can be programmably micro-adjusted; for example, the jitter control condition is implemented by randomly displacing the microdose pixel from the true location of the target cone in the pixel array. In 3 of 20 color matching sessions, the jitter control is implemented by splitting each microdose into four, displaced to the corners of a square at two cone spacings away from the target cone. Rasterization and FPGA data transmission also comprehensively account for the system’s spatiotemporal properties, including: computational latency of eye tracking and data transmission, sinusoidal scanning velocity of the resonant mirrors, and timing offsets in individual PMT outputs and AOM inputs relative to a common clock.

#### Transverse chromatic aberration compensation

Transverse chromatic aberration (TCA) causes the different wavelength channels of the laser beam to refract to different lateral locations on the retina. The effect is that the pixel rasters for each laser wavelength differ in offset and magnification, often by several cones. Measuring TCA translation and magnification enables simple geometric compensation in the rasterization step of the previous section. We improve on previous image-based procedures (*46*) for measuring TCA, which are based on simultaneous reflection imaging of all wavelength channels by interlacing lines for each channel in the raster stream. The NCC algorithm is used to spatially align each of the resulting videos, with the required translation being the required TCA measurement.

The key challenge that we improve upon is high noise in video signals for visible channels due to low reflected photon counts at the 543 and 488 nm PMTs. We find the noise is too high in 488 for successful NCC-based alignment. Fortunately, the infrared video usually has excellent signal-to-noise ratio. Our improvement is to modify the R-SLAM algorithm (*42*) to process the interlaced video recordings, using the infrared image to spatially stabilize and remove motion distortions from all interlaced channels including the noisy ones, and generating low-noise retinal maps for all wavelength channels by temporal averaging of the stabilized and undistorted video frames. We further extend the prior work to solve for both translation and scaling components of TCA between the different wavelength channels.

We also computationally verify the correctness of TCA measurement and compensation before beginning experiments. We draw a fiducial mark in each interlaced channel, by zeroing out the pixels for the fiducial in all channels using TCA compensation. The resulting retina videos for each channel will show the fiducial as black pixels in the retina imagery; correct TCA is verified by spatially aligning the cone imagery in the different channels and confirming that the fiducial marks in all channels are simultaneously aligned. We use the R-SLAM method to generate low-noise retina images in all channels, as described, and by drawing a grid of fiducials to visually verify TCA compensation across the entire field of view.

TCA is highly dependent on pupil location (*46*). We position the subject’s head precisely via a bitebar and use a pupil tracker and manual bitebar position adjustments to maintain the same pupil position during experiments as during TCA measurement.

### Color matching experiments

#### Color matching tools

The RGB projector that we use for color matching is a DLP LightCrafter projector (Wintech, Carlsbad, CA), which is coaligned with the AOSLO’s optical path. We control this display using the Psychophysics Toolbox (*47*–*49*). The tunable-laser source that we use for color matching is a SuperK supercontinuum laser connected to the SuperK VARIA tunable filter (NKT Photonics, Birkerød, Denmark) that allows interactive selection of the spectral bandwidth and center wavelength.

Our custom color matching control panel is a Behringer X-Touch MINI USB Controller (Behringer, Willich, Germany), which we programmed specifically for the color matching task. In experiments using the RGB projector, subjects use three free-wheeling dials on this controller to adjust the hue, saturation, and value of the projector-generated matching square and an additional dial to add desaturating white light to the Oz square if necessary to achieve an overall match. In experiments using the tunable-wavelength source, subjects use two dials to adjust the center wavelength and overall intensity of the matching square, and have the option of using two additional dials to add desaturating white light either to the stimulus or the matching field. We use a bandwidth of 10 nm, and the center wavelength can be adjusted over a range from 405 to 525 nm.

#### Subject view

**Fig. 5.**
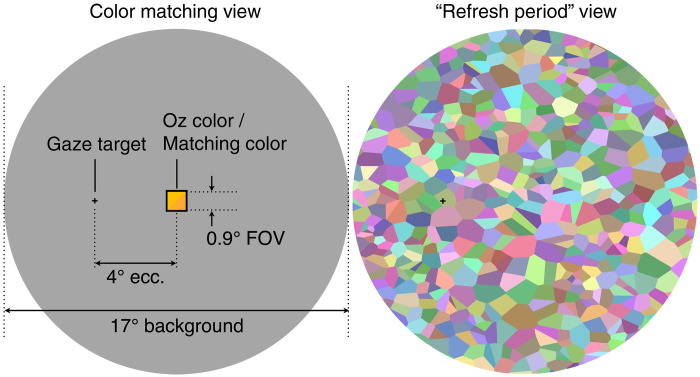
Subject’s view during color matching experiment. Left shows the experimental view. Right shows an example of the multicolored mosaics shown for a periodic 15-s “refresh period.”

A sketch of the subject’s view during color matching is shown in Fig. 5. The projector displays a gaze target that we instruct subjects to fixate on for the duration of the experiment. Approximately 4° adjacent to the gaze target, there is a 0.9° square in which both the Oz color and matching color appear in alternation. This geometry ensures that the AOSLO stimulates the classified region of the subject’s retina.

The projector generates a gray background field that is 17° in diameter. The chromaticity of this gray background is taken as the reference white point for plotting in Fig. 3. The luminance of this gray background is kept at approximately 500 cd/m^2^ to establish photopic light levels and avoid interference from rod activation. The gray background pixels are turned off within the Oz/matching square so that no projector light is added to either stimulus. This black square is 1.2× the size of the stimulus, so that any slight misalignment between the AOSLO and projector display does not cause the stimuli to mix with the gray background.

#### Color matching protocol

During a color matching trial, subjects adjust the controllable color until they achieve a match with the Oz color. The Oz and matching squares of color are spatially coincident and alternate in time, turning on for 1 s each followed by 1 s of darkness in a repeating cycle. Subjects are free to take as much time as necessary to achieve a match. Every 30 s, there is a “refresh period” during which the entire field is replaced with a series of multicolored mosaics inspired by the “wipeout” pattern from prior work in hue matching under fixation (*11*). Each mosaic appears for 1 s, and each refresh period lasts 15 s. This period is intended to mitigate the effect of afterimages, which diminish the apparent color saturation of the matching field and Oz stimulus. To submit a match, subjects must have undergone this refresh period within 12 s prior and not made any further color adjustments. This is to ensure that the submitted match can be quickly confirmed while the presence of afterimages is minimal.

As an option, subjects can view the Oz and matching squares vertically side by side, synchronously alternating 2-s on and 2-s off. This allows simultaneous comparison of the two colors, but subjects must return to the spatially overlapped and temporally alternating view to judge the match using a common patch of adapted retina before submission.

Subjects may note that the color and luminance of the stimulus is not uniform across the entire field of stimulation. In particular, some parts of the field may occasionally appear as the color of the stimulating laser itself, regardless of the LMS activation levels being targeted. This is because any errors in delivery will tend to cause the percept to skew toward the color of the stimulating laser. Subjects are told to match to the color they see that is most dissimilar from the natural color of the laser.

#### Plotting color matches

To compute LMS color match coordinates, we spectrally characterize the RGB projector and the tunable laser using a spectroradiometer [PR-650, PhotoResearch (now Jadak), North Syracuse, NY]. We measure the spectral power distribution for the projector’s red, green, and blue pixel primaries and the linearity of each primary across its 8-bit range, subject to a lookup table, which we compute in a calibration step. We compute color match coordinates as the weighted sum of the red, green, and blue primary spectra, each scaled according to the measured output levels corresponding to the matching RGB values selected by the subject. For matches made using the tunable laser system, we individually measure the spectrum of each match setting that was submitted during the experiment. Any match involving both the projector’s desaturating white and the tunable-wavelength source can be constructed by combining the spectra for the two sources. All color match average points are computed in 3D LMS coordinates before projection into *lms* chromaticity space.

The projector’s gamut is the triangle enclosed by the chromaticity coordinates of its red, green, and blue primaries, as shown in Fig. 3 (A and B). The tunable laser’s gamut is the edge of the spectral locus corresponding to wavelengths between 405 and 525 nm. When combined with white light from the projector, its overall gamut for matching is encompassed by the region outlined in Fig. 3C. To match colors beyond the boundary of these gamuts, the subject adds an amount of projector white to desaturate the Oz color until a color match is achievable. The resulting coordinates for the Oz color are the coordinates of this desaturating white subtracted from the coordinates of the matching color.

**Fig. 6.**
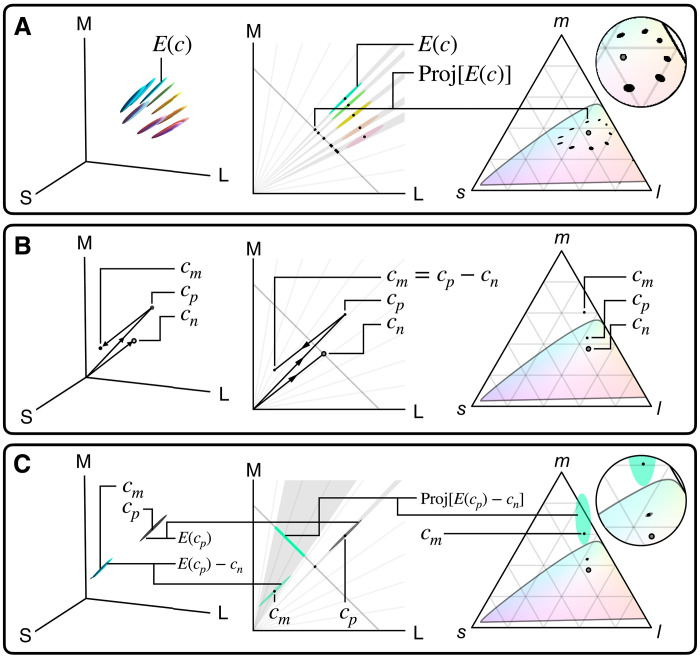
Computation of perceptual uncertainty ellipses. (**A**) Human perceptual JND ellipsoids [e.g., *E*(*c*) at color *c*] are long and skinny, pointing at the origin in LMS Cartesian space. They project to ellipses in the $\left(l,m,s\right)=\frac{\left(L,M,S\right)}{L+M+S}$ chromaticity triangle as shown. (**B**) Vector math for computing the coordinates of a color match *c*_*m*_ = *c*_*p*_ − *c*_*n*_, where *c*_*p*_ is the “positive” color seen by subject at matching, and *c*_*n*_ is the “negative” light added to the test color to enable a match. (**C**) In color matching, because the color seen by the subject at the time of match submission is the “positive” color *c*_*p*_, the perceptual uncertainty of the inferred color match *c*_*m*_ is the ellipsoid *E*(*c*_*p*_), recentered on *c*_*m*_, as shown. If *c*_*n*_ is non-negative, as shown, the ellipsoid recentered on *c*_*m*_, *E*(*c*_*p*_) − *c*_*n*_, no longer points at the origin, and projects to a nonlinearly enlarged ellipse in the *lms* triangle. Therefore, it is desirable to minimize the “negative” light required to achieve a color match, as accomplished with the tunable monochromatic light source used for matching olo (see Fig. 3).

In Figs. 2, 3, and 6, we draw the natural human gamut as reference, using colors that approximately convey how those chromaticities would appear to test subjects, by mapping (*l*, *m*, *s*) to sRGB (*50*) colors with matching CIELAB hue (*51*).

Care must be exercised in comparing the relative positions of color match points and the boundary of the human gamut. Plotting both of these relies on standard Stockman-Sharpe cone spectral response functions. The exact cone response functions for subjects are unknown, but in general, individual cone responses will vary from the standard, for example, due to photopigment optical density. Using variations in the assumed cone responses during plotting will cause small geometric shifts in the spectral locus and color match points, and it is possible for color match points in Fig. 3 (A and B) near the boundary to fall outside or inside the gamut depending on the cone responses assumed. In contrast, note that the color match points in Fig. 3C always fall outside the human gamut, because subjects compare olo against near-monochromatic colors that lie on the boundary of the gamut regardless of the cone responses assumed in plotting.

#### Plotting perceptual uncertainty in matching

In Fig. 3, we plot the relative perceptual uncertainty of color matches as ellipses around the color coordinates. These ellipses are proportional in size to the perceptually just-noticeable-difference (JND) region of colors surrounding the matching color. Notably, these perceptual ellipses in Fig. 3 are small for orange matches and relatively large for colors that are outside the color matching system’s gamut, like olo. It turns out that this phenomenon is entirely due to two factors that we explain in this section: the natural shape of human perceptual uncertainty ellipsoids in 3D colorspace and the vector geometry of calculating chromaticities from color matching results. Figure 6 illustrates the phenomenon.

First, human color discrimination ability varies across colorspace as first quantified by MacAdam (*52*) and codified in standards such as CIELAB and ${\Delta E}_{\mathrm{ab}}^{*}$ (*51*). We use the CIELAB standard to estimate the relative size of JND spheres [${\Delta E}_{\mathrm{ab}}^{*}=2.3$ (*53*)], then transform them into LMS colorspace, where they become ellipsoids. It is a feature of human perception that these ellipsoids are long and skinny, pointing toward the origin of LMS space as shown in Fig. 6A. Since plotting *lms* chromaticity coordinates involves projection through the origin [$(l,m,s)=\frac{\left(L,M,S\right)}{L+M+S}$], these ellipsoids project to relatively small 2D error ellipses as shown on the triangle in Fig. 6A. Let us denote the JND ellipsoid at a given color *c*_*o*_ in LMS coordinates as *E*(*c*_*o*_).

Second, the vector geometry of calculating color matches is shown in Fig. 6B. Let us denote the inferred LMS color coordinates of the test color as *c*_*m*_ = *c*_*p*_ − *c*_*n*_, where *c*_*p*_ represents the LMS coordinates of the “positive” color actually seen by the subject when submitting the color match result, and *c*_*n*_ represents the LMS coordinates of the “negative” light added by the subject to the test color. A critical observation is that the perceptual uncertainty of a color match is defined by human color discrimination power around *c*_*p*_, the “positive” color seen at submission, not the test color *c*_*m*_. To prove this, note that when the color match is submitted, the human subject is looking at colors *c*_*p*_ and (*c*_*m*_ + *c*_*n*_) in alternation, and judging them to be perceptually a match. Any color within *E*(*c*_*p*_), the perceptual JND ellipsoid at *c*_*p*_, would have been perceptually indistinguishable and also considered a match at submission. Therefore, the perceptual uncertainty ellipsoid for a color matching result is *E*(*c*_*p*_) centered at *c*_*m*_ = *c*_*p*_ − *c*_*n*_, as shown in Fig. 6C.

Last, when *c*_*n*_ is nonzero, the vector shift of the long, skinny ellipsoid means that it is no longer oriented toward the origin in LMS colorspace. Therefore, projecting toward the origin to compute the *lms* chromaticity will cause the long axis of the ellipsoid to spread into a larger ellipse on the chromaticity diagram. This effect grows nonlinearly as *c*_*n*_ increases, as shown in Fig. 6C.

To recap, the ellipses drawn in Fig. 3 are simply the relative perceptual uncertainty around the color match coordinate computed by the process above, drawn three times the actual size of a projected JND ellipsoid computed by the CIELAB/Δ*E* standard. The shape of these ellipses depends only on the position of the subject’s average match location and are not derived from the spread of the matches themselves. That is, they account for potential noise in matching due to the perceptual characteristics of color space near the match, but do not reflect other sources of variation, such as individual observer differences or system performance variations from trial to trial.

Note that CIELAB is defined with respect to an assumed white point. In our calculations, the white point has the chromaticity matching the gray background field (Fig. 5) and the CIE Y luminance of either the stimulus or the background, whichever is greater.

#### Qualitative hue naming and saturation rating

In addition to color matching, we carry out color naming experiments in which subjects observe a stimulus, then name its hue and rate its saturation on a scale from 1 to 4. Subjects see the same fixation cross and gray background, with stimuli appearing at 4° eccentricity from fixation, as in the color matching experiments. Stimuli turn on and off for 2 s each on a continuous cycle, and a refresh period occurs before each new stimulus is shown. Subjects are instructed to observe the stimulus for as long as necessary, then to report their qualitative description.

### Image and video recognition experiments

In the 4-AFC and 2-AFC tasks, we aim to eliminate any luminance cues that could allow subjects to detect line orientation or motion direction without using hue information. In particular, because our subjects have more L cones than M cones, a higher density of microdoses are directed at the foreground object than the background in AFC stimuli, which would create a difference in luminance between them. Thus, in a pre-experiment calibration step, subjects adjust the intensity of microdoses directed at the L cones while viewing a jittered moving disk stimulus until the field appears equiluminant. This selected intensity level is then used to render any L-targeted microdoses in the subsequent experiments.
