# Supplementary material for: Novel color via stimulation of individual photoreceptors at population scale
Source: Sci Adv. 2025 Apr 18;11(16):eadu1052. doi: 10.1126/sciadv.adu1052 (PMC12007580; doi:10.1126/sciadv.adu1052)
Supplement: Supplementary file 1 — Legend for movie S1 [file sciadv.adu1052_sm.pdf]

Science Advances
AAAS

Supplementary Materials for

Novel color via stimulation of individual photoreceptors at population scale

James Fong *et al.*

Corresponding author: Ren Ng, ren@berkeley.edu

Sci. Adv. **11**, eadu1052 (2025)
DOI: 10.1126/sciadv.adu1052

The PDF file includes:

Legend for movie S1

Other Supplementary Material for this manuscript includes the following:

Movie S1

Movie S1.

This video gives a general overview of the work and describes each figure in the paper in order. It includes animations to aid in the understanding of each figure, along with examples of real retinal videos and laser microdose recordings.
